# Genetic Signatures of Selection for Cashmere Traits in Chinese Goats

**DOI:** 10.3390/ani10101905

**Published:** 2020-10-18

**Authors:** Meilin Jin, Jian Lu, Xiaojuan Fei, Zengkui Lu, Kai Quan, Yongbin Liu, Mingxing Chu, Ran Di, Huihua Wang, Caihong Wei

**Affiliations:** 1 Key Laboratory of Animal Genetics and Breeding and Reproduction of Ministry of Agriculture, Institute of Animal Sciences, Chinese Academy of Agricultural Sciences, Beijing 100193, China; jmlingg@163.com (M.J.); 18409481571@163.com (X.F.); mxchu@263.net (M.C.); dirangirl@163.com (R.D.); 2 National Animal Husbandry Service, Beijing 100193, China; lujian34@163.com; 3 Lanzhou Institute of Husbandry and Pharmaceutical Sciences, Chinese Academy of Agricultural Sciences, Lanzhou 730050, China; luzengkui@caas.cn; 4 Institute of Animal Science and Technology, Henan University of Animal Husbandry and Economy, Zhengzhou 450046, China; quankai1115@163.com; 5 Inner Mongolia Academy of Animal Husbandry Science, Hohhot 010031, China; ybliu117@126.com

**Keywords:** cashmere trait, *Fst*, XP-EHH, cashmere goat

## Abstract

**Simple Summary:**

Cashmere goats are a unique husbandry resource in China. These goats are well known for producing the highest cashmere yield and best fiber quality in the world. Although cashmere is highly valued and also known as “fiber gem” and “soft gold”, few studies have examined the genetic basis of cashmere traits in cashmere goats. Here, we identified selection signals by comparing Fst and XP-EHH (the cross population extend haplotype homozygosity test) of a non-cashmere breed (Huanghuai goat) with those of two cashmere breeds (Inner Mongolia and Liaoning cashmere goats). Two genes (WNT10A and CSN3) were potentially associated with cashmere traits. This information may be valuable for studying the genetic uniqueness of cashmere goats and elucidating the mechanisms underlying cashmere traits in cashmere goats.

**Abstract:**

Inner Mongolia and Liaoning cashmere goats in China are well-known for their cashmere quality and yield. Thus, they are great models for identifying genomic regions associated with cashmere traits. Herein, 53 Inner Mongolia cashmere goats, Liaoning cashmere goats and Huanghuai goats were genotyped, and 53,347 single-nucleotide polymorphisms (SNPs) were produced using the Illumina Caprine 50K SNP chip. Additionally, we identified some positively selected SNPs by analyzing *Fst* and XP-EHH. The top 5% of SNPs had selection signatures. After gene annotation, 222 and 173 candidate genes were identified in Inner Mongolia and Liaoning cashmere goats, respectively. Several genes were related to hair follicle development, such as *TRPS1*, *WDR74*, *LRRC14*, *SPTLC3, IGF1R*, *PADI2*, *FOXP1*, *WNT10A* and *CSN3*. Gene enrichment analysis of these cashmere trait-associated genes related 67 enriched signaling pathways that mainly participate in hair follicle development and stem cell pluripotency regulation. Furthermore, we identified 20 overlapping genes that were selected in both cashmere goat breeds. Among these overlapping genes, *WNT10A* and *CSN3*, which are associated with hair follicle development, are potentially involved in cashmere production. These findings may improve molecular breeding of cashmere goats in the future.

## 1. Introduction

Cashmere goats produce cashmere [1] from the secondary hair follicle [2,3]. Cashmere is finer, softer, lighter, and more insulating than sheep wool; thus, it is considered a luxury fiber [4,5,6]. Cashmere goats are only found in specific areas of Asia [7]. In China, there are more than 20 native Cashmere goat breeds that yield 75% of the Cashmere produced worldwide [8]. Among these breeds, Inner Mongolia and Liaoning Cashmere goats are well known for the thin fiber diameter and high yield of their Cashmere [9]. Therefore, researchers have attempted to identify the underlying genetic mechanisms to produce higher Cashmere quality and yield. 

With the development of high-throughput technology, association studies between genetic diversity and phenotypes can be carried out at the genome level. Selection of a trait could be traced by detecting selection signatures in a genome, and the relevant genes can be identified by selection signal analysis [10]. Recently, several preliminary studies have attempted to reveal the natural and artificial selection footprint in Cashmere goat breeds. Su et al. evaluated the primary and secondary hair follicle transcriptome of the Inner Mongolia Cashmere goats. Several genes have been identified that may regulate the Cashmere growth phases, including eight Interleukin-17 receptor B and Zinc-finger protein [4]. Wang et al. used medium-coverage (9-13x) sequences from eight domesticated goat breeds to identify genomic regions. Three genes related to Cashmere trait (*LHX2*, *FGF9* and *WNT2*) were identified [11]. Guan et al. used the whole genome sequence from six Cashmere goat breeds and six non-Cashmere goat breeds to identify candidate genes can improving fiber traits (e.g., *FGF5*) [12]. Li et al. performed selective signal analysis on the resequencing data of 70 Cashmere goats and 14 non-Cashmere goats, and identified some genes that are potentially involved in Cashmere fiber production (*FGF5*, *SGK3*, *IGFBP7*, *OXTR* and *ROCK1*) [13]. Despite these useful findings, research on genetic mechanisms underlying Cashmere production is still insufficient. Therefore, it is important to study the biological characteristics of any genes that may be involved in regulating Cashmere growth [14].

Here, we compared Inner Mongolia and Liaoning Cashmere goats with the Huanghuai goats. Based on *Fst* and Cross Population Extend Haplotype Homozygosity Test (XP-EHH) analyses, relevant genes correlated with Cashmere traits were identified based on selection signals. *Fst*, which is based on allele frequency difference, can identify candidate genes between populations, and it is widely used because of its simplicity [15]. XP-EHH detect candidate regions based on haplotypes among populations, and is implemented along with *Fst* to eliminate false positives and improve signal analysis accuracy [16]. Therefore, we used *Fst* and XP-EHH to detect genes and merged the genes to identify the strongest selection signature by *Fst* and XP-EHH. These finding will provide a further theoretical basis for research on the formation mechanism of Cashmere and protection of local goat resources. Additionally, some important genes related to Cashmere traits were identified that can be used to enrich the gene pool of candidate Cashmere goats. 

## 2. Materials and Methods

### 2.1. Biological Sample Collection and Genotyping

All experimental procedures involving goats were approved by the Science Research Department (in charge of animal welfare issue) of the Institute of Animal Sciences, Chinese Academy of Agricultural Sciences (IAS-CAAS) (Beijing, China). Ethical approval on animal survival was given by the animal ethics committee of IAS-CAAS (No. IASCAAS-AE-03, 12 December 2016). In this study, 53 individuals of three goat breeds were collected from conservation farms or core areas of origin and including 17 Inner Mongolia Cashmere goats (MGR), 20 Liaoning Cashmere goats (LNR) and 16 Huanghuai goats (HHG) (Table 1). The Cashmere goat breed probably originated from the *Capra falconeri* [17]. Moreover, three other representative goat breeds were included to assess goat breed relationships; we included 15 Daiyun goats (DYG), 16 Chengdu Brown goats (CDM) and 15 Longlin goats (LLG). All specimens were arbitrarily selected females, and all specimens were healthy and did not have any gastrointestinal disease. Genomic DNA samples were obtained by blood collection from the jugular vein, and the TIAN amp Blood DNA Kit (Tian gen Biotech Co. Ltd., Beijing, China) was used to extract genomic DNA. A Nanodrop 2000 nucleic acid protein analyzer (Thermo, Scientific, Wilmington, NC, USA) was used to measure the genomic DNA purity and concentration. After removing the low-quality DNA samples, standard quality samples were genotyped using the Illumina Caprine SNP 50K Chip, which contains 53,347 single-nucleotide polymorphisms (SNPs).

### 2.2. Quality Control

To increase the data processing accuracy, stringent quality control criteria were applied. SNPs were removed if they could not pass the following four criteria: (i) Hardy_Weinberg equilibrium *p* < 10^−6^; (ii) SNP call rate > 90%, (iii) SNP minimum allele frequency (MAF) > 0.01 and (iv) presence of mapped autosomal loci.

### 2.3. Population Structure Analysis

Principal component analysis (PCA) was performed using PLINK1.90 to discern genetic relationships among breeds [18]. The graphical representation of PCA was depicted using plot function in R 3.6.1 (https://www.rdocumentation.org/packages/ggplot2) [19]. A neighbor-joining (N-J) tree was constructed using VCF 4.2 and tassel 4.3 (https://sourceforge.net/projects/tassel/) [20]. The N-J tree was illuminated and visualized by iTOLv4 (https://itol.embl.de/upload.cgi) [21]. In addition, ADMIXTURE 1.30 was also implemented to reveal admixture patterns among these six goat breeds [22]. The corresponding cross-validation error value for clustering (K = 2 to 3) was also calculated in ADMIXTURE. The solutions for each K-value were visualized using Excel 2010 [23]. 

### 2.4. Selection Signals Analysis

To detect the potential mechanisms underlying Cashmere traits, we searched for autosomal SNPs that showed evidence of specific selection by comparing *Fst* and XP-EHH values between the Cashmere and non-Cashmere goats detecting selection signals among genome-wide SNP genotypes [24,25]. We compared the reference population (HHG) with two Cashmere goat breeds (LNR and MGR) to obtain *Fst* and XP-EHH values. Two approaches were used to identify selection signatures in the domestic goat genomes. First, the genomic regions with strongly differing or differentially fixed variants in alleles frequency between different breeds were identified by the population differentiation index (*Fst)*, which is the conventional measure of population genetic differentiation. We calculated *Fst* value for each SNP as *Fst* = $\frac{MSP-MSG}{MSP+\left(n_{c}-1\right)MSG}$, where *MSP* is the mean square error within the populations, *MSG* represents the mean square error between the two populations, and *n_c_* represents the average sample size of the entire population after correction [26,27].

Furthermore, we also computed the XP-EHH values using haplotype information in the XP-EHH program [28] to determine the positive selection in MGR and LNR. Haplotypes were estimated with fastphase 1.4 [29]. We used population label information to estimate phased haplotype background and the following options for each chromosome: -Ku40 -Kl10 -Ki10. Specifically, XP-EHH values of individual SNPs were first calculated based on $EHH\left(x\right)=\frac{\sum_{i=1}^{hx}\left(\frac{ni}{2}\right)}{\left(\frac{n_{a}}{2}\right)+\left(\frac{n_{A}}{2}\right)},XP-EHH=\left(\frac{\int_{D}^{}EHH_{pop1^{\left(x\right)dx}}}{\int_{D}^{}EHH_{pop2^{\left(x\right)}}dx}\right)$, where *n_a_* and *n_A_* are the numbers of alleles a and A haplotypes, respectively; *n_i_* is the number of the ith haplotype in a population, and *hx* represents the number of different haplotypes in a genomic region at locus X. The integral domain D (the cutoff value of the X integral) was selected, so that the EHH values of both populations were reduced to sufficiently small values [30].

### 2.5. Gene Annotation and Gene Enrichment Analysis

We defined candidate selection SNPs as those that fell into the upper 95th percentile of the empirical distribution with extremely high *Fst* and XP-EHH values (top 5% level). Based on the goat reference genome annotation, the 50-kb upstream and downstream regions of significant SNP loci were operationally defined as candidate regions under selection. Genes were identified by these different methods and annotated using the goat genome information database. Gene function was determined using the National Center for Biotechnology Information database (https://www.ncbi.nlm.nih.gov/) and by literature search. We selected some significant genes associated with Cashmere trait submitted to the DAVID database (http://david.abcc.ncifcrf.gov/) for Kyoto Encyclopedia of Genes and Genomes (KEGG) analysis [31]. The selected background organism was *Capra hircus*. Significantly enriched pathway was indicated by Q-value < 0.05.

## 3. Results

### 3.1. Genetic Variation and Population Genetic Analysis

**Figure 1:**
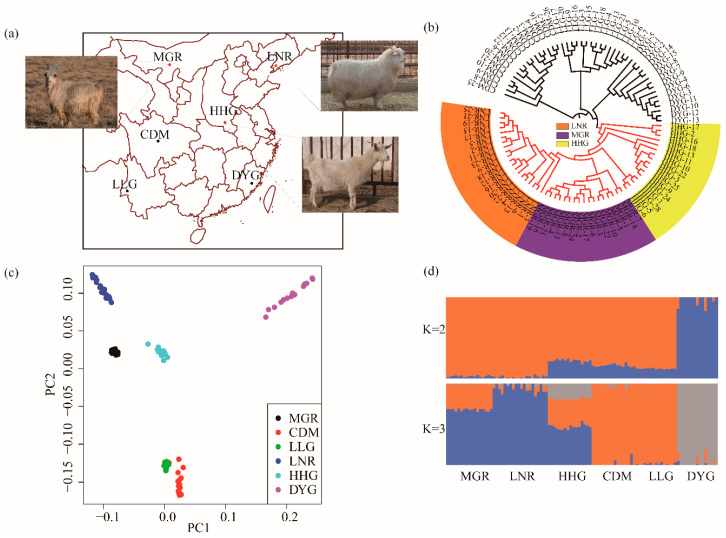
(**a**) Geographic map indicating the distribution of the Cashmere goats sampled in this study (Photographs were reference from Animal Genetic resources in China sheep and goats [8]). Map of sample locations in the present study. The map was created using the R package “map tools”, URL: http://rforge.r-project.org/projects/maptools/. (**b**) Neighbour-joining (NJ) phylogenetic tree for the six breeds based on pairwise Fst. Each population is represented by a different colour and symbol label: Liaoning Cashmere goats are indicated in red; Inner Mongolia Cashmere goats are indicated in orange and Huanghuai goats are yellow. (**c**) Plots for the first (Component 1) and second (Component 2) dimensions revealed the clustering of six breeds (Inner Mongolia Cashmere goat, Chengdu goat, Longlin goat, Liaoning Cashmere goat, Huanghuai goat and Daiyun goat). (**d**) Population structure of six goat breeds inferred by ADMIXTURE results from K = 2–3 are shown.

After data quality control, 45,575 SNP markers were passed through filters and quality control. The individuals were representative of six native goat breeds in China: MGR, LNR, HHG, DYG, CDM and LLG (north to south) (Figure 1a). According to the N-J tree, the phylogenetic relationship of the six breeds revealed genetically distinct clusters, and LNR, HHG and MGR were located on the same distinct branch (Figure 1b). This is consistent with a report that MGR and LNR came from a recent common origin [32]. This result was also similar to the finding of another previous study [33]. The genetic relationships among the six Chinese native goat breeds revealed by PCA are shown in Figure 1c. The first dimension (PC1) clearly separated MGR and LNR from the others breeds. The second dimension (PC2) separated MGR, LNR, HHG and DYG from the other two breeds. PC1 and PC2 showed that MGR, LNR and HHG clusters grouped closest to each other (Figure 1c). The PCA results showed a similar pattern as the N-J tree plot. For ADMIXTURE analysis, which was conducted for ancestry estimation [22], when K = 3, there was a division between MGR, LNR, HHG and other breeds (Figure 1d).

### 3.2. Detecting Positive Selection Genes

**Figure 2:**
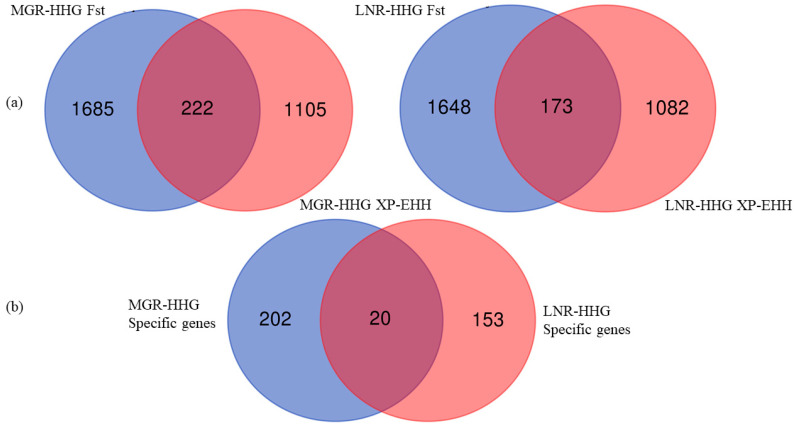
(**a**) Venn diagrams of each breed s selection genes from XP-EHH and *Fst* approaches; (**b**) Venn diagram of specific selection genes in three breeds.

*Fst* revealed a high coefficient of genetic differentiation, and has been widely used to identify selection signals among whole-genome SNPs [28,34]. We calculated the *Fst* for each SNP and ultimately annotated 1907 (*Fst* > 0.2878) and 1821 genes (*Fst* > 0.3248) in MGR and LNR, respectively. XP-EHH was also calculated for each SNP [24,28]; the top 5% of the XP-EHH values, which were consider candidate selection SNPs, included 1327 and 1255 genes in MGR and LNR, respectively. We merged the gene lists generated by these two approaches and identified 222 (MGR-HHG) and 173 (LNR-HHG) specific genes that showed the strongest selection signatures (Figure 2a). Finally, there were also 20 genes that overlapped between the two Cashmere breeds (Figure 2b). 

### 3.3. Specific Selection Genes in Each Cashmere Breed

**Figure 3:**
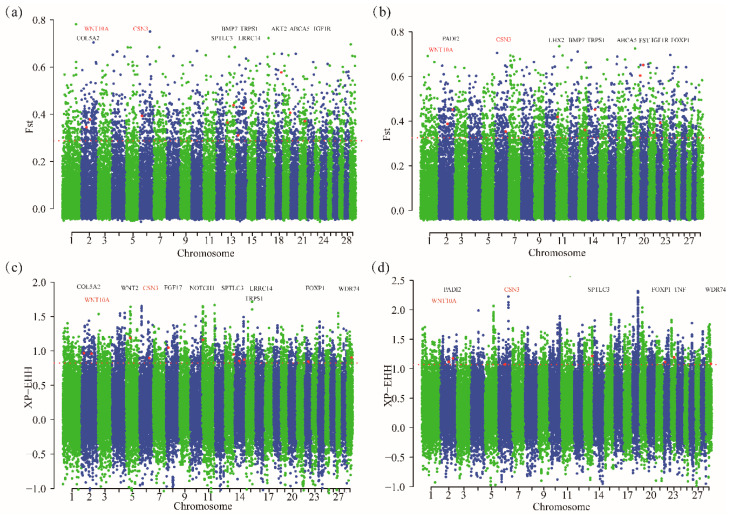
Genome-wide distribution of *FST* and XP-EHH values. red dots represent sites showing significant signal in the *Fst* approach or XP-EHH approach. (**a**) Inner Mongolia Cashmere goat by *Fst* values. (**b**) Liaoning Cashmere goat breed by *Fst* values. (**c**) Inner Mongolia Cashmere goat by XP-EHH values. (**d**) Liaoning Cashmere goat breed by XP-EHH values.

Here, we identified many candidate genes in different Cashmere goat breed (Table 2). In MGR, *COL5A2*, *WNT10A*, *CSN3*, *SPTLC3*, *LRRC14*, *TRPS1* and *WDR74* were associated with Cashmere traits. *PADI2 WNT10A*, *CSN3*, *IGF1R* and *FOXP1* were identified in LNR (Figure 3).

### 3.4. KEGG Pathway Enrichment Analysis of Important Genes in Cashmere Goat

In our study, Cashmere trait-related genes were selected for KEGG enrichment analysis and mainly showed clustering within follicle-related pathways (Q-value < 0.05). The KEGG pathway analysis revealed that the genes associated with the significantly correlated SNPs were highly enriched in 67 pathways (Q-value < 0.05, Supplementary Table S1). Further analysis indicated that these pathways included the signaling pathways that regulate stem cells pluripotency, basal cell carcinoma, TGF-beta signaling, melanogenesis, Wnt signaling, TNF signaling and PI3K-Akt signaling (Supplement Figure S1).

## 4. Discussion

Detection of selection signals associated with economic traits has been successfully implemented in several livestock species, such as cattle [35], pigs [36], sheep [37] and horses [38]. However, many approaches have been proposed to identify selection signals in genomes, such as Fst, XP-EHH and iHS. In this study, the SNPs identified by high *Fst* and XP-EHH values produced a subset of 18 SNPs, which represented 20 genes (Supplementary Table S2). The genes that overlapped between the two approaches were putatively defined as candidate genes with strong selection signatures. Of these genes, *WNT10A* and *CSN3* have plausible biological functions associated with Cashmere traits. *WNT10A* is a member of the WNT gene family [39,40,41,42]. WNT paracrine signaling molecules belong to a family of conserved glycoproteins with at least 19 members in humans and mice [42]. Additionally, the WNT pathway is considered the master regulator during hair follicle morphogenesis [43]. WNT signaling proceeds through EDA/EDAR/NF-kappa B signaling. NF-kappa B regulates the WNT pathway and acts as a signal mediator by upregulating Shh ligand expression [44,45]. Moreover, *WNT10A* plays an important role in hair follicle development [46]. Thomas et al. [47] demonstrated that a primary WNT (*WNT3*) and secondary WNTs (*WNT0A* and *WNT10B*) are essential for hair follicle initiation, morphogenesis and development. *CSN3*(casein kappa) represents one of the most important proteins that determine the manufacturing properties of milk [48]. However, *CSN3* has other plausible biological functions related to Cashmere traits, and may be involved in regular hair follicle development [49,50]. *STAT5* activation acts as a mesenchyme switch to trigger natural anagen entry in post-developmental hair follicle cycling. *STAT5* deletion results in significantly upregulated expression of genes, such as *CSN3*, *Dkk3* and *Dlk1*, which inhibit WNT and Notch signaling. Alternatively, *WNT6*, *FGF7* and *FGF10*, known regulators of anagen induction/progression, were all significantly downregulated [49,50]. Therefore, we inferred that *CSN3* also has an important role in hair follicle development, although the precise function of *CSN3* relative to Cashmere traits remains unknown and should be verified in future studies. 

In mammals, coat hair protects against environmental changes [11]. Mohair is produced in the primary and secondary hair follicles [51]. However, the coat hair of Cashmere goats differs from mohair, because Cashmere is produced by the secondary hair follicle and coarse hair is produced by the primary hair follicle. Additionally, Cashmere yield is the main economic trait of Cashmere goats. There is now clear evidence that associated gastrointestinal parasitic status of goats with characteristics of their hair, like *Diarrhoea*, which is a major impediment to profitable hair goat production in many countries as it predisposes animals to blowfly strike and contaminates wool [52]. *Haemonchus contortus* is a bloodsucking parasite that causes an extreme stress on animals that are already protein deficient owing to the requirements for hair growth, especially in hair goat. Ectoparasites also can inflict serious damage in the hair and pelts of goats, such as *Bovicola (Damalinia) caprae*, the biting louse of goats, which is the most serious external parasite in hair goats. Economic losses are realized by hair damage occurring when goats bite or rub themselves to relieve itching [53]. In addition, Cashmere is also influenced by hair follicle depth and density, which have high heritability [54]. Genetically, the size and number of cells in the Cashmere tissue also determines the differences in Cashmere growth. Cashmere production by goat hair follicles is related to epidermal hair cells change, which indicates that this periodic change is related to the change in the epidermis, but not dermal thickness [55]. Candidate genes that were correlated with the hair follicle were identified in this study. *PADI2* is predominantly in skeletal muscle and belongs to PAD, which is responsible for the formation of protein-bound citrulline, a major amino acid in the inner root sheath and hair follicle medulla [56]. *FOXP1* is crucial for maintaining quiescence of hair follicle stem cells. *FOXP1* loss in skin epithelial cells leads to precocious stem cell activation, which results in a drastic shortening of the quiescent phase of the hair cycle [57]. In addition, hair growth regulation mainly depends on the cyclic activities of the hair follicle and the successive catagen and telogen phases in the growth cycles [6]. A previous study found that hair growth of Inner Mongolia Cashmere goats was regulated by the hypertrichosis gene *TRPS1* [12], which is similar to our study outcomes. Moreover, many hair follicle-related genes were identified in this study. For example, *WDR74* and *WNT10A* were related to hair follicle morphogenesis [43], and *LRRC14* regulates hair follicle induction [45] and inhibits NF-kB activation [58]; therefore, these genes may regulate hair follicle induction and are important for hair follicle and epidermal appendage development [59]. 

In general, the secondary follicle cycle can be divided into three periods: anagen (April–November), regression (December–January the following year), and telogen (from February to March the following year) [60]. In the anagen phase, Cashmere rapidly grows under the control of related genes [6]. The anagen phase can be further divided into early (April_August) and flourishing anagen phases (August_November). Early research on mouse hair follicles showed that the ability of the secondary hair follicle cycle to enter the growth stage was limited to the early anagen phase; once the secondary hair follicle cycle entered the flourishing anagen phase, the ability to return to the early anagen phase was lost [61]. The transition between early and flourishing growth was regulated by the expression of many inhibitors, such as bone morphogenetic proteins (BMPs) [61,62]. Interestingly, in our study, *IGF1R* was identified in Liaoning Cashmere goats; this gene regulates the anagen phase by activating *BMP4*, which affects the stem cell bulge and hair follicle cycle [63]. Moreover, pioneering research demonstrated that exogenously supplied *FGF18* can prevent the hair follicle stem cells of *FOXP1* null mice from being prematurely activated. As *FGF18* controls telogen phase length and is a key downstream target of *FOXP1*, *FOXP1* regulates the telogen stem cell state in the hair follicle stem cell niche by controlling *FGF18* expression [57]. *TRPS1* directly represses expression of the hair follicle stem cell regulator *Sox9* to control follicle epithelium proliferation [64], and plays an important role in hair follicle cycling [65].

KEGG pathway analysis revealed that some important pathways were remarkably enriched for Cashmere traits. The hair follicle is a unique mini organ that self-renews throughout its lifetime. Many stem cell populations reside within human hair follicles and enable their regeneration [66]. Stem cell pluripotency plays a crucial role in cell fate decisions by controlling self-renewal and differentiation [44,67]. The Wnt signaling pathway is up-regulated in the telogen phase and peaks in the anagen phase, which is considered a key factor in stimulating hair growth in dermal papilla cells [68]; therefore, this pathway plays an essential role in hair follicle induction [44]. The Wnt signalling pathway has emerged as a potential regulator of hematopoietic stem cell self-renewal [69]. The PI3K/Akt signaling pathway is essential for de novo hair follicle regeneration [70]. Moreover, the role of melatonin in promoting Cashmere yield has been demonstrated for decades [71], and melatonin also participates in stem cell differentiation pathways. Melatonin induces Cashmere growth through melatonin receptors [72] and is a critical intermediary between photoperiod and Cashmere growth [73]. Cashmere goats breed seasonally, and usually give birth between March and April, which conforms to a spring kidding system [72]. Melatonin is administered to Cashmere goats in April, because melatonin treatment in adult Cashmere goats during the Cashmere non-growth period has been found to induce Cashmere growth, increase Cashmere yield, improve Cashmere quality and decrease the fiber diameter [74], which also present similar results from other countries of the world [75]. In Australian Cashmere-bearing feral goats, sustained immunization against melatonin gave rise to two growth cycles, resulting in a mean increase in Cashmere production of 78% over controls in the first year [76].

## 5. Conclusions

We conducted a comprehensive study to identify selection signatures in Cashmere and non-Cashmere goats based on Illumina Caprine 50K SNP chip data. Our results showed that a high degree of genetic diversity was present in our goat samples, and many important genes and pathways related to Cashmere traits could be targeted for trait improvement. We found that *CSN3* may have a novel selection signal associated with goat Cashmere production. However, no clear main effect genes for Cashmere growth have been found. Our research enriches the candidate gene pool for elucidating the mechanisms underlying Cashmere traits and provides an important basis for protecting and sustainably using Cashmere goats. However, further study is needed to fully understand Cashmere production in Inner Mongolia and Liaoning Cashmere goats.

## Figures and Tables

**Table 1:** Information of the Chinese goat populations in this study.

Breeds	Abbr.	Sample Size	Sex	Breed Characteristics	Location	Cashmere Yield (g) [8]	Fiber Diameter (μm) [8]	Weight (kg) [8]
Inner Mongolia Cashmere goats	MGR	17	female	multipurpose	Erdos, Inner Mongolia, China	623 ± 86.32	15.2 ± 1.10	29.9 ± 3.0
Liaoning Cashmere goat	LNR	20	female	multipurpose	Wafangdian, Liaoning, China	641 ± 145	15.5 ± 0.77	43.2 ± 2.6
Huanghuai	HHS	16	female	multipurpose	Zhumadian, Henan, China	0	-	37.8 ± 7.4
Daiyun goats	DYG	15	female	meat	Quanzhou, Fujian, China	0	-	30.5 ± 5.0
Chengdu Brown goat	CDM	16	female	multipurpose	Chengdu, Sichuan, China	0	-	39.1 ± 6.6
Longlin goats	LLG	15	female	meat	Longlin, Guangxi Autonomous Region, China	0	-	33.7 ± 5.1

**Table 2:** The information of main important genes for Cashmere goats.

Breed	Chromosome	Position (bp)	Fst	XP-EHH	Candidate Gene
MGR	2	7,312,610	0.345233	0.963794	*COL5A2*
	2	106,272,304	0.377815	0.956545	*WNT10A*
	6	82,906,006	0.393008	0.892872	*CSN3*
	13	6,058,455	0.364966	0.946052	*SPTLC3*
	14	11,106,589	0.310467	0.868854	*LRRC14*
	14	59,346,613	0.427461	0.851539	*TRPS1*
	29	39,094,189	0.466096	0.899931	*WDR74*
LNR	2	134,768,458	0.451128	1.17724	*PADI2*
	2	106,213,716	0.387751	1.11715	*WNT10A*
	6	82,906,041	0.353381	1.07532	*CSN3*
	21	5,892,765	0.348628	1.2051	*IGF1R*
	22	30,284,836	0.391823	1.11317	*FOXP1*

## Supplementary Materials

The following are available online at https://www.mdpi.com/2076-2615/10/10/1905/s1, Figure S1: KEGG analysis for the regional candidate genes with genome-wide significant association, Table S1: KEGG analysis for the regional candidate genes with genome-wide significant association, Table S2: Overlapping selection candidate genes for Cashmere goats by *Fst* and XP-EHH.

## References

[1] Liu Y., Wang L., Li X., Han W., Yang K., Wang H., Zhang Y., Su R., Liu Z., Wang R. (2017). High-throughput sequencing of hair follicle development-related micrornas in cashmere goat at various fetal periods. Saudi J. Biol. Sci..

[2] Dai B., Zhang M., Yuan J.L., Ren L.Q., Han X.Y., Liu D. (2019). Integrative Analysis of Methylation and Transcriptional Profiles to Reveal the Genetic Stability of Cashmere Traits in the Tβ4 Overexpression of Cashmere Goats. Animals.

[3] Gao Y., Wang X., Yan H., Zeng J., Ma S., Niu Y., Zhou G., Jiang Y., Chen Y. (2016). Comparative Transcriptome Analysis of Fetal Skin Reveals Key Genes Related to Hair Follicle Morphogenesis in Cashmere Goats. PLoS ONE.

[4] Su R., Fan Y., Qiao X., Li X., Zhang L., Li C., Li J. (2018). Transcriptomic analysis reveals critical genes for the hair follicle of Inner Mongolia cashmere goat from catagen to telogen. PLoS ONE.

[5] Watkins P., Buxton A. (1992). Luxury Fibres: Rare Materials for Higher Added Value. https://agris.fao.org/agris-search/search.do?recordID=GB19940084324.

[6] Liu B., Gao F., Guo J., Wu D., Hao B., Li Y., Zhao C. (2016). A Microarray-Based Analysis Reveals that a Short Photoperiod Promotes Hair Growth in the Arbas Cashmere Goat. PLoS ONE.

[7] Di R., Vahidi S.M.F., Ma Y.H., He X.H., Zhao Q.J., Han J.L., Guan W.J., Chu M.X., Sun W., Pu Y.P. (2010). Microsatellite analysis revealed genetic diversity and population structure among Chinese cashmere goats. Anim. Genet..

[8] Du L.X. (2011). Animal Genetic Resources in China.

[9] Wang L., Peng L.Q., Zhang W.B., Zhang Z.Y., Yang W.Y., Ding L., Tang S.M., Wu H.Y. (1996). Initation and development of skin follicles in the Inner Mongolia white cashmere goat. Acta Vet. Zootech. Sin..

[10] Horscroft C., Ennis S., Pengelly R.J., Sluckin T.J., Collins A. (2018). Sequencing era methods for identifying signatures of selection in the genome. Brief. Bioinform..

[11] Wang X., Liu J., Zhou G., Guo J., Yan H., Niu Y., Li Y., Yuan C., Geng R., Lan X. (2016). Whole-genome sequencing of eight goat populations for the detection of selection signatures underlying production and adaptive traits. Sci. Rep..

[12] Guan D., Luo N., Tan X., Zhao Z., Huang Y., Na R., Zhang J., Zhao Y.-J. (2016). Scanning of selection signature provides a glimpse into important economic traits in goats (*Capra hircus*). Sci. Rep..

[13] Li X., Su R., Wan W., Zhang W., Jiang H., Qiao X., Fan Y., Zhang Y., Wang R., Liu Z. (2017). Identification of selection signals by large-scale whole-genome resequencing of cashmere goats. Sci. Rep..

[14] Jin M., Zhang J.-Y., Chu M.X., Piao J., Piao J.-A., Zhao F.-Q. (2018). Cashmere growth control in Liaoning cashmere goat by ovarian carcinoma immunoreactive antigen-like protein 2 and decorin genes. Asian Austral. J. Anim. Sci..

[15] Yuan Z., Liu E., Liu Z., Kijas J.W., Zhu C., Hu S., Ma X., Zhang L., Du L., Wang H. (2016). Selection signature analysis reveals genes associated with tail type in Chinese indigenous sheep. Anim. Genet..

[16] Ma Y., Wei J., Zhang Q., Chen L., Wang J., Liu J., Ding X. (2015). A Genome Scan for Selection Signatures in Pigs. PLoS ONE.

[17] Bai W. (2009). Molecular Characterization of Male and Female Origin and Genetic Differentiation in Chinese Cashmere Goat Breeds. Ph.D. Thesis.

[18] Purcell S., Neale B., Todd-Brown K., Thomas L., Ferreira M.A.R., Bender D., Maller J., Sklar P., De Bakker P.I.W., Daly M.J. (2007). PLINK: A Tool Set for Whole-Genome Association and Population-Based Linkage Analyses. Am. J. Hum. Genet..

[19] Chan B.K.C. (2018). Data Analysis Using R Programming. Adv. Exp. Med. Biol..

[20] Subramanian S., Ramasamy U., Chen D. (2019). VCF2PopTree: A client-side software to construct population phylogeny from genome-wide SNPs. PeerJ.

[21] Letunic I., Bork P. (2019). Interactive Tree of Life (iTOL) v4: Recent updates and new developments. Nucleic Acids Res..

[22] Alexander D.H., Novembre J., Lange K. (2009). Fast model-based estimation of ancestry in unrelated individuals. Genome Res..

[23] Divisi D., Di Leonardo G., Zaccagna G., Crisci R. (2017). Basic statistics with Microsoft Excel: A review. J. Thorac. Dis..

[24] Sabeti P.C., Schaffner S.F., Fry B., Lohmueller J., Varilly P., Shamovsky O., Palma A., Mikkelsen T.S., Altshuler D., Lander E.S. (2006). Positive Natural Selection in the Human Lineage. Science N. Y..

[25] Pickrell J.K., Coop G., Novembre J., Kudaravalli S., Li J.Z., Absher D., Srinivasan B.S., Barsh G.S., Myers R.M., Feldman M.W. (2009). Signals of recent positive selection in a worldwide sample of human populations. Genome Res..

[26] Akey J.M., Zhang G., Zhang K., Jin L., Shriver M.D. (2002). Interrogating a High-Density SNP Map for Signatures of Natural Selection. Genome Res..

[27] Jin M., Lu J., Fei X., Lu Z., Quan K., Liu Y., Chu M., Di R., Wei C., Wang H. (2020). Selection Signatures Analysis Reveals Genes Associated with High-Altitude Adaptation in Tibetan Goats from Nagqu, Tibet. Animals.

[28] Wei C., Wang H., Liu G., Zhao F., Kijas J.W., Ma Y., Lu J., Zhang L., Cao J., Wu M. (2016). Genome-wide analysis reveals adaptation to high altitudes in Tibetan sheep. Sci. Rep..

[29] Scheet P., Stephens M. (2006). A Fast and Flexible Statistical Model for Large-Scale Population Genotype Data: Applications to Inferring Missing Genotypes and Haplotypic Phase. Am. J. Hum. Genet..

[30] Sabeti P.C., Varilly P., Fry B., Lohmueller J., Hostetter E., Cotsapas C., Xie X., Byrne E.H., McCarroll S.A., Gaudet R. (2007). Genome-wide detection and characterization of positive selection in human populations. Nat. Cell Biol..

[31] Huang D.W., Sherman B.T., Lempicki R.A. (2009). Systematic and integrative analysis of large gene lists using DAVID bioinformatics resources. Nat. Protoc..

[32] Li C., Yin J., Zhang Y. (2005). Comparative Study on Skin and Hair Follicles Cycling between Inner Mongolia and Liaoning Cashmere Goats. Acta Vet. Zootech. Sin..

[33] Wei C., Lu J., Xu L., Liu G., Wang Z., Zhao F., Zhang L., Han X., Du L., Liu C. (2014). Genetic Structure of Chinese Indigenous Goats and the Special Geographical Structure in the Southwest China as a Geographic Barrier Driving the Fragmentation of a Large Population. PLoS ONE.

[34] Bruno W.J., Socci N.D., Halpern A.L. (2000). Weighted Neighbor Joining: A Likelihood-Based Approach to Distance-Based Phylogeny Reconstruction. Mol. Biol. Evol..

[35] Cheruiyot E.K., Bett R.C., Amimo J.O., Zhang Y., Mrode R., Mujibi F.D.N. (2018). Signatures of Selection in Admixed Dairy Cattle in Tanzania. Front. Genet..

[36] Diao S.-Q., Huang S., Chen Z., Teng J., Ma Y., Yuan X., Chen Z.-M., Zhang H., Li J.-Q., Zhang Z. (2019). Genome-Wide Signatures of Selection Detection in Three South China Indigenous Pigs. Genes.

[37] Manzari Z., Mehrabani-Yeganeh H., Nejati-Javaremi A., Moradi M.H., Gholizadeh M. (2019). Detecting selection signatures in three Iranian sheep breeds. Anim. Genet..

[38] Ablondi M., Viklund Å., Lindgren G., Eriksson S., Mikko S. (2019). Signatures of selection in the genome of Swedish warmblood horses selected for sport performance. BMC Genom..

[39] Kelly G.M., Lai C.-J., Moon R.T. (1993). Expression of Wnt10a in the Central Nervous System of Developing Zebrafish. Dev. Biol..

[40] Wang J., Shackleford G.M. (1996). Murine Wnt10a and Wnt10b: Cloning and expression in developing limbs, face and skin of embryos and in adults. Oncogene.

[41] Kirikoshi H., Sekihara H., Katoh M. (2001). WNT10A and WNT6, Clustered in Human Chromosome 2q35 Region with Head-to-Tail Manner, Are Strongly Coexpressed in SW480 Cells. Biochem. Biophys. Res. Commun..

[42] Kimura R., Watanabe C., Kawaguchi A., Kim Y.-I., Park S.-B., Maki K., Ishida H., Yamaguchi T. (2015). Common polymorphisms in WNT10A affect tooth morphology as well as hair shape. Hum. Mol. Genet..

[43] He N., Dong Z., Tai D., Liang H., Guo X., Cang M., Dongjun L. (2018). The role of Sox9 in maintaining the characteristics and pluripotency of Arbas Cashmere goat hair follicle stem cells. Cytotechnology.

[44] Rishikaysh P., Dev K., Diaz D., Qureshi W.M.S., Filip S., Mokry J. (2014). Signaling Involved in Hair Follicle Morphogenesis and Development. Int. J. Mol. Sci..

[45] Zhang Y., Wang L., Li Z., Chen D., Han W., Wu Z., Shang F., Hai E., Wei Y., Su R. (2019). Transcriptome profiling reveals transcriptional and alternative splicing regulation in the early embryonic development of hair follicles in the cashmere goat. Sci. Rep..

[46] Sulayman A., Tian K., Huang X., Tian Y., Xu X., Fu X., Zhao B., Wu W., Wang D., Yasin A. (2019). Genome-wide identification and characterization of long non-coding RNAs expressed during sheep fetal and postnatal hair follicle development. Sci. Rep..

[47] Andl T., Reddy S.T., Gaddapara T., Millar S.E. (2002). WNT Signals Are Required for the Initiation of Hair Follicle Development. Dev. Cell.

[48] Reale S., Yahyaoui M.H., Folch J.M., Sànchez A., Pilla F., Angiolillo A. (2005). Genetic polymorphism of the K-casein (CSN3) gene in goats reared in Southern Italy. Ital. J. Anim. Sci..

[49] Shin H.Y., Hennighausen L., Yoo K.H. (2018). STAT5-Driven Enhancers Tightly Control Temporal Expression of Mammary-Specific Genes. J. Mammary Gland. Biol. Neoplasia.

[50] Legrand J.M.D., Roy E., Ellis J.J., François M., Brooks A.J., Khosrotehrani K. (2016). STAT5 Activation in the Dermal Papilla is Important for Hair Follicle Growth Phase Induction. J. Investig. Dermatol..

[51] Nazari-Ghadikolaei A., Mehrabani-Yeganeh H., Miarei-Aashtiani S.R., Staiger E.A., Rashidi A., Huson H.J. (2018). Genome-Wide Association Studies Identify Candidate Genes for Coat Color and Mohair Traits in the Iranian Markhoz Goat. Front. Genet..

[52] Williams A.R., Palmer D.G. (2012). Interactions between gastrointestinal nematode parasites and diarrhoea in sheep: Pathogenesis and control. Vet. J..

[53] Bretzlaff K. (1990). Special Problems of Hair Goats. Vet. Clin. N. Am. Food Anim. Pract..

[54] Li G.F., Zhao Z.Z., Li D.Q., Guo H.Y. (2002). The observation of derma structure and control of wool growth. Grass Feed. Livest..

[55] Jin H.G., Zhang B.H. (1995). Study on skin hair follicle structure and seed selection method of Inner Mongolia white cashmere goat. J. Beijing Agric. Univ..

[56] Rogers G., Winter B., McLaughlan C., Powell B., Nesci T. (1997). Peptidylarginine Deiminase of the Hair Follicle: Characterization, Localization, and Function in Keratinizing Tissues. J. Investig. Dermatol..

[57] Leishman E., Howard J.M., Garcia G.E., Miao Q., Ku A.T., Dekker J.D., Tucker H., Nguyen H. (2013). Foxp1 maintains hair follicle stem cell quiescence through regulation of Fgf18. Development.

[58] Wu C., Yang Y., Ou J., Zhu L., Zhao W., Cui J. (2016). LRRC14 attenuates Toll-like receptor-mediated NF-κB signaling through disruption of IKK complex. Exp. Cell Res..

[59] Zheng Y.Y., Sheng S.D., Hui T.Y., Yue C., Sun J.M., Guo D., Guo S.L., Li B.J., Xue H.L., Wang Z.Y. (2019). An Integrated Analysis of Cashmere Fineness lncRNAs in Cashmere Goats. Genes.

[60] Bai W.L., Yin R.H., Yin R.L., Wang J.J., Jiang W.Q., Luo G.B., Zhao Z.H. (2013). IGF1mRNA Splicing Variants in Liaoning Cashmere Goat: Identification, Characterization, and Transcriptional Patterns in Skin and Visceral Organs. Anim. Biotechnol..

[61] Plikus M.V., Mayer J.A., De La Cruz D., Baker R.E., Maini P.K., Maxson R., Chuong C.-M. (2008). Cyclic dermal BMP signalling regulates stem cell activation during hair regeneration. Nat. Cell Biol..

[62] Plikus M.V., Baker R.E., Chen C.-C., Fare C., De La Cruz D., Andl T., Maini P.K., Millar S.E., Widelitz R., Chuong C.-M. (2011). Self-Organizing and Stochastic Behaviors During the Regeneration of Hair Stem Cells. Science N. Y..

[63] Castela M., Linay F., Roy E., Moguelet P., Xu J., Holzenberger M., Khosrotehrani K., Aractingi S. (2017). Igf1rsignalling acts on the anagen-to-catagen transition in the hair cycle. Exp. Dermatol..

[64] Chisholm A.D., Fantauzzo K.A., Kurban M., Levy B., Christiano A.M. (2012). Trps1 and Its Target Gene Sox9 Regulate Epithelial Proliferation in the Developing Hair Follicle and Are Associated with Hypertrichosis. PLoS Genet..

[65] Lawler J. (2007). Anti-Angiogenic Therapy with Thrombospondins. Tumor Angiogenes.

[66] Ohyama M. (2019). Use of human intra-tissue stem/progenitor cells and induced pluripotent stem cells for hair follicle regeneration. Inflamm. Regen..

[67] Suda T., Arai F. (2008). Wnt Signaling in the Niche. Cell.

[68] Gentile P., Garcovich S. (2019). Advances in Regenerative Stem Cell Therapy in Androgenic Alopecia and Hair Loss: Wnt Pathway, Growth-Factor, and Mesenchymal Stem Cell Signaling Impact Analysis on Cell Growth and Hair Follicle Development. Cells.

[69] Reya T., Clevers H. (2005). Wnt signalling in stem cells and cancer. Nat. Cell Biol..

[70] Chen Y., Fan Z., Wang X., Mo M., Zeng S.B., Xu R.-H., Wang X., Wu Y. (2020). PI3K/Akt signaling pathway is essential for de novo hair follicle regeneration. Stem Cell Res. Ther..

[71] Ge W., Wang S.-H., Sun B., Zhang Y.-L., Shen W., Khatib H., Wang X. (2018). Melatonin promotes Cashmere goat (*Capra hircus*) secondary hair follicle growth: A view from integrated analysis of long non-coding and coding RNAs. Cell Cycle.

[72] Yang C.H., Wu Z.Y., Li Y., Zhang W. (2019). Effect of melatonin administration to lactating cashmere goats on milk production of dams and on hair follicle development in their offspring. Animal.

[73] Wang L.F., Yang G.Q., Yang Y.S., Zhang S.J., Wang Y.L., De-Xun L.U. (2008). Effects of Photoperiod and Melatonin on Endocrine and Cashmere Growth in Cashmere Goats in Telogen. China Anim. Husb. Vet. Med..

[74] Duan C., Xu J., Sun C., Jia Z., Zhang W. (2015). Effects of melatonin implantation on cashmere yield, fibre characteristics, duration of cashmere growth as well as growth and reproductive performance of Inner Mongolian cashmere goats. J. Anim. Sci. Biotechnol..

[75] Foldes A., Hoskinson R., Baker P.J., McDonald B., Maxwell C., Restall B. (1992). Effect of immunization against melatonin on seasonal fleece growth in feral goats. J. Pineal Res..

[76] Mustafa F.E.-Z.A., Abdel-Maksoud F.M., Hassan A.H.S., Mokhtar D.M. (2020). Melatonin induces a stimulatory action on the scrotal skin components of Soay ram in the non-breeding season. Sci. Rep..

